# Voltage-Dependent Electronic Transport Properties of Reduced Graphene Oxide with Various Coverage Ratios

**DOI:** 10.1007/s40820-014-0017-1

**Published:** 2014-10-31

**Authors:** Serhan Yamacli

**Affiliations:** Department of Electrical-Electronics Engineering, Nuh Naci Yazgan University, 38090 Kayseri, Turkey

**Keywords:** Reduced graphene oxide, Coverage ratio, Negative differential resistance

## Abstract

Graphene is mainly implemented by these methods: exfoliating, unzipping of carbon nanotubes, chemical vapour deposition, epitaxial growth and the reduction of graphene oxide. The latter option has the advantage of low cost and precision. However, reduced graphene oxide (rGO) contains hydrogen and/or oxygen atoms hence the structure and properties of the rGO and intrinsic graphene are different. Considering the advantages of the implementation and utilization of rGO, voltage-dependent electronic transport properties of several rGO samples with various coverage ratios are investigated in this work. Ab initio simulations based on density functional theory combined with non-equilibrium Green’s function formalism are used to obtain the current–voltage characteristics and the voltage-dependent transmission spectra of rGO samples. It is shown that the transport properties of rGO are strongly dependent on the coverage ratio. Obtained results indicate that some of the rGO samples have negative differential resistance characteristics while normally insulating rGO can behave as conducting beyond a certain threshold voltage. The reasons of the peculiar electronic transport behaviour of rGO samples are further investigated, taking the transmission eigenstates and their localization degree into consideration. The findings of this study are expected to be helpful for engineering the characteristics of rGO structures.

## Introduction

Graphene is an atomic-thick layer made of carbon atoms in honeycomb lattice structure [1]. Graphene was firstly isolated by means of mechanical exfoliating by Novoselov and Geim in [2], and since then studies regarding graphene and graphene based devices are taking great interest. The main reasons of the interest in graphene stem from ultra-high electron mobility [3], ballistic electron transport and the lowest resistivity at room temperature [4]. These properties of graphene make it possible to utilize graphene as interconnects [5], channel regions of graphene nanoribbon field effect transistors (GNRFETs) [6], anode materials in high-efficiency batteries [7] and transparent conductor applications [8]. From an implementation point of view, graphene is basically produced in these ways: chemical vapour deposition (CVD) [9], epitaxial growth [10], exfoliation [11], unzipping of carbon nanotubes (CNTs) and the reduction of graphene oxide (GO) [12]. Exfoliation is a mechanical method, firstly used by Geim and Novoselov, in which single or few layers of graphene is cleaved from a stack of hundreds of thousands of graphene layers. On the other hand, in some recent studies, it is shown that chemical unzipping of CNTs can lead to seamless graphene layers with narrow widths [13]. However, for a cheaper implementation, GO, which is known for more than a century [14], is reduced to conduct reduced graphene oxide (rGO) by chemical [15], thermal [16] or photonic methods [17, 18]. The widely method used for the reduction of GO to graphene is the chemical method where GO is treated by hydrazine [19]. Although this is a well-established method, the drawback is that the distribution of oxygen and hydrogen content of the resulting graphene cannot be accurately controlled. The thermal reduction involves heating GO directly in the furnace or using microwaves [20]; however, the drawback is that the crystal structure of the rGO is also affected by heating. For a cost-effective and accurately controlled reduction, photonic reduction seems the best alternative as (i) it can directly be applied on the material where rGO will be used, (ii) it is cost-effective to use a light source compared to chemical methods and (iii) the percentage of the carbon atoms having bonds with contamination atoms in a rGO crystal, namely the coverage ratio [21], can be controlled accurately and position-dependently.

The reduction of GO to rGO is a low cost method for obtaining graphene; however, the main drawback is that the rGO is not an exact graphene sheet but a relatively imperfect honeycomb crystal with controllable oxygen and hydrogen content. Moreover, the atomic structure and the chemical formula of GO and rGO is still under debate [4, 21, 22]. Several models for GO are proposed in the literature such as Hoffmann model [23], Nakajima-Matsuo model [24], Ruess model [25], Scholz-Boehm model [26], Zsabo-Dekany model [27] and Lerf-Klinowski model [28]. For a detailed overview of these chemical models, the reader can be referred to [4] and [29]. From an application viewpoint, the relationship between the chemical structure and properties of rGO is of great importance thus extensively considered and discussed in the literature which can be summarized as follows: Mu et al. studied the thermal transport in rGO and concluded that thermal conductivity of rGO is highly dependent on the oxygen coverage [30]. Paci et al. investigated the atomic structure of rGO and they have shown that hydroxyl and epoxide groups may be positioned at any location of the structure [31]. Room-temperature conductances of various rGO samples are experimentally studied by Gomez-Navarro et al., and it is shown that increased reduction of GO leads to an increased conductance [32]. Gilje et al. implemented rGO sheets and concluded that rGO includes OH groups and may show semiconductor behaviour depending on the coverage percentage [33]. Hirata et al. thermally reduced GO to rGO and have shown that the reduction can lead to a conductivity up to 1,600 S m^−1^ [34]. Similarly, Stankovich et al. demonstrated that chemical reduction of GO increases the conductivity [35]. Zhang et al. studied rGO with oxygen adatoms and have concluded that it is possible to open a band gap with increasing the O/C ratio [3]. Similarly, Yan and Chou have shown that epoxy and hydroxyl groups tend to aggregate on rGO and the band gap of rGO can be tuned by the percentage of epoxy and hydroxyl groups [36]. A similar result is achieved also by Yan et al. [37]. On the other hand, the atomic configurations of rGO clearly have to be studied carefully in order to investigate the electronic transport properties of GO and rGO structures. For example, Kudin et al. studied the Raman spectra of rGO samples and reported various rGO configurations with hydroxyl and epoxy groups [38]. Similarly, Casabianca et al. investigated the locations of O and H atoms on rGO structures using multidimensional ^13^C solid-state nuclear magnetic resonance [39]. Xu and Xue have utilized density functional theory (DFT) calculations in order to show that inclusion of epoxy groups alter the lattice constant of rGO samples [40]. Among these studies, Boukhalov and Katsnelson have given a comprehensive analysis of the atomic structure and the density of states (DOS) characteristics of rGO structures where the conductance properties of rGO dependent on the coverage percentage are investigated. In their detailed analysis, the following points are agreed: (i) most stable configuration of rGO is with 75 % coverage, (ii) rGO with a coverage higher than 25 % includes both hydroxyl and epoxy groups, (iii) the reduction of GO under 6.25 % coverage ratio is difficult and (iv) rGO is expected to be conducting for coverages less than 25 % according to DOS plots [21].

Although the conductance properties of various rGO configurations are investigated in the literature as summarized above, the conductance characteristics are estimated using the obtained band gaps and DOS characteristics which show zero-bias behaviour. However, it is recently shown that the conductance characteristics of functionalized graphene structures have to be evaluated by taking the voltage-dependent transmission spectra and voltage-dependent transmission eigenstates into consideration [41] which is missing in the literature for rGO structures. Considering the utilization of rGO in electronic applications, analyses of their voltage-dependent electronic transport characteristics are clearly essential. Therefore, in this study, the voltage-dependent electronic transport behaviours of realistic rGO samples with various coverage ratios are obtained utilizing first-principles simulations. The current–voltage characteristics are interpreted taking the possible application areas of the rGO structures into consideration. Obtained results show that rGO structures with different coverage ratios have peculiar electronic transport characteristics.

## Materials and Methods

### First-Principles Simulations of Nano Devices

There are various methods used for analysing the characteristics and behaviours of nano-scaled structures in the literature such as tight-binding calculations [42], semi-empirical approach [43] and DFT simulations [44]. Among these procedures, DFT simulations provide proven results thanks to the fact that Schrodinger’s equation is solved numerically with exchange-correlation functionals which model the electron–electron and electron–ion interactions accurately. DFT is a widely-used method in ab initio simulations giving precise results for a broad range of nano devices such as CNTs [45], graphene [46] and molecular devices [47]. Various parameters such as electron density, DOS and total energy of nano structures can be obtained utilizing DFT. Moreover, electronic transport properties including the transmission spectra, current density and transmission eigenvalues can also be obtained using DFT when utilized in conjunction with non-equilibrium Green’s function (NEGF) formalism [48]. Computationally, DFT and NEGF are combined in transiesta method [49]. In this study, analyses of the transport properties of various rGO samples are aimed hence a package capable of applying the transiesta method is required. Therefore, Atomistix Toolkit (ATK^®^) program based on the transiesta method is utilized for obtaining the transport properties of the rGO structures considered in this paper.

### Investigated Reduced Graphene Oxide Structures

As it is stated before, rGO structures with more than 25 % coverage are expected to have both hydroxyl and oxygen contamination while the conducting rGO sheets with 25 % or less coverage contain hydroxyl groups only. Self-consistent energy calculations show that for rGO structures with more than 25 % coverage, chemisorption energy is 60 and 30 meV lower per hydroxyl and oxygen, respectively, compared to configurations having same type of contaminations of the same coverage ratios [21]. Moreover, in general, oxygen bonds with two carbon atoms while hydroxyl group bonds with a single carbon atom on each face of graphene structure. On the other hand, GO can be reduced to rGO from 50 % coverage to 6.25 % coverage by several steps. Considering this situation, the transport properties of GO structures with 50, 25, 12.5 and 6.25 % coverage ratios are studied. The reason of selecting the lower limit of coverage ratio as 6.25 % is that the reduction of GO down to this coverage is practical [21].

**Fig. 1:**
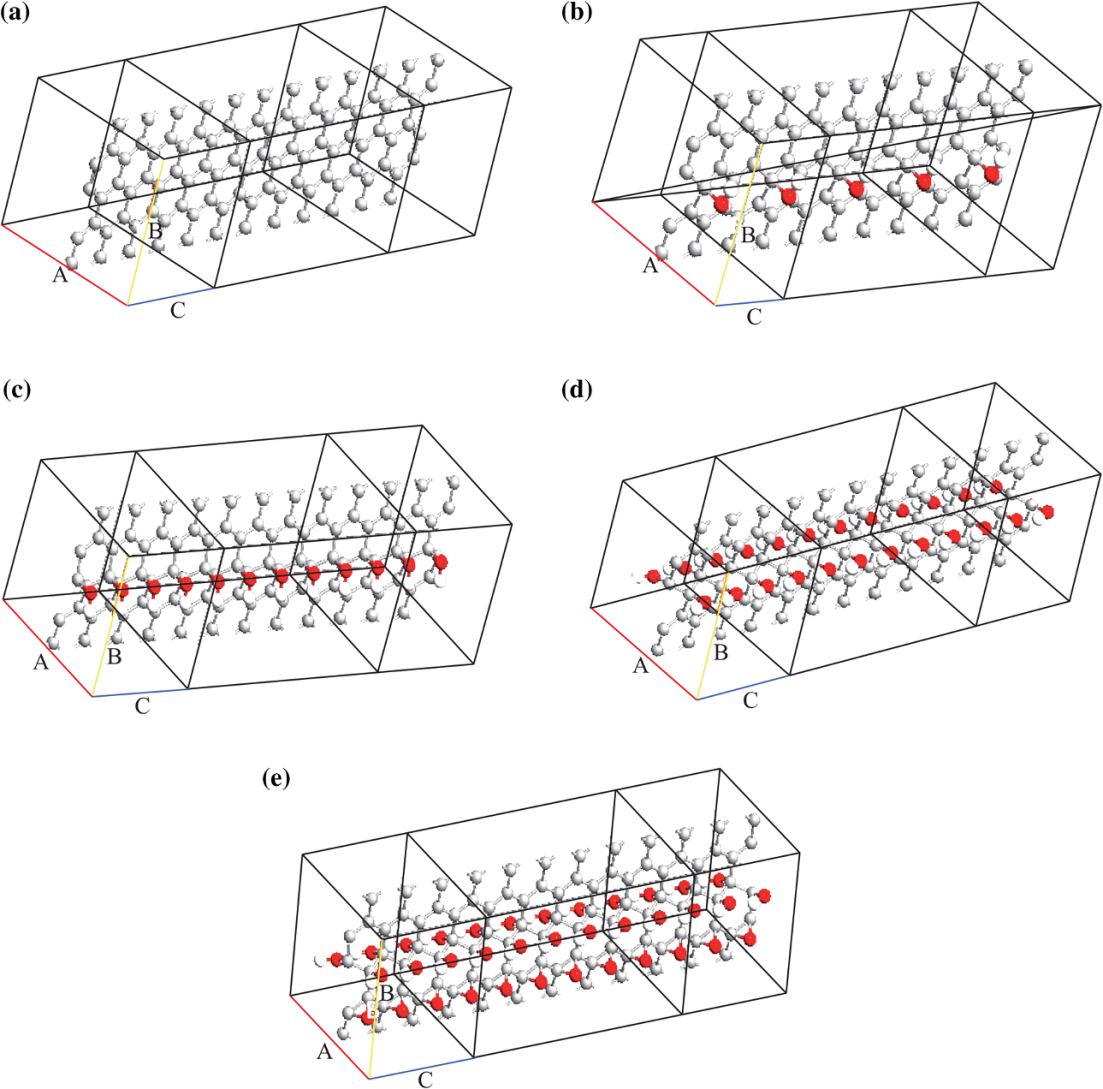
Considered rGO and intrinsic graphene structures: **a** Intrinsic graphene sheet, **b**–**e** rGO structures with 6.25, 12.5, 25 and 50 % coverages, respectively. *Grey*, *red* and *white balls* represent carbon, oxygen and hydrogen atoms, respectively. (Color figure online)

 The unit cells of the considered rGO structures are built in ATK with the approximate oxygen and hydroxyl group positions [21]. These geometries of these structures are then optimized in ATK^®^ with DFT method using the following parameters: mesh cut-off is 150 Ry, electron temperature is 300 K, k-point sampling is (1,11,11) and the exchange-correlation functional is local density approximation (LDA) which gives accurate results for carbon-based structures [50]. It is worth noting that coordinates of the optimized structures can be found on the web version of this paper. The optimization procedure is completed for each of the rGO unit cells when the force on each atom is less than 0.05 eV Å^−1^. After the optimization step, six periods of scattering region for each rGO sample are built with the optimized structures. The reason for selecting six periods of scattering region is that it is enough to avoid electrode interactions while providing a balanced simulation cost [51]. As a reference configuration, an intrinsic graphene sheet is also built in ATK as shown in Fig. 1a where rGO structures with 6.25, 12.5, 25 and 50 % coverage ratios are shown in Fig. 1b–e, respectively. Voltage-dependent electronic transport properties of the considered structures are obtained for the 0–1 V voltage range. The reason of selecting this voltage range is the estimations of the international technology roadmap for semiconductors [52].

## Results and Discussion

**Fig. 2:**
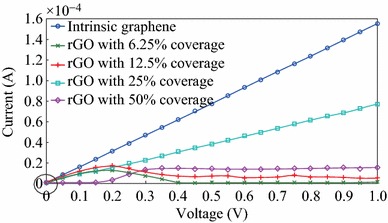
Current-voltage variations of the intrinsic graphene sheet and rGO samples with various coverage percentages

Firstly, the current–voltage variations of the intrinsic graphene sheet and the rGO samples with different coverage ratios are obtained in ATK^®^ for 21 applied voltage points in the 0–1 V range as shown in Fig. 2. The first observation considering these *I*–*V* characteristics is that at zero-bias; rGO with 50 % coverage shows non-conducting characteristics, while rGO samples with coverage ratios of 25 % and lower clearly show conducting characteristics as shown inside the circle. These results are in accordance with [21]. However, the goal of our study is that the voltage-dependent electronic transport behaviours of these rGO samples are obtained for the 0–1 V applied voltage range and not only for the zero-bias state. Investigation of the *I*–*V* behaviour in this range shows peculiar properties as explained below.

The *I*–*V* behaviour of the rGO sheet with 25 % coverage shows a linear trend implying that the transmission eigenchannels of 25 % coverage rGO is maintained in the 0–1 V range. Another important result is that rGO samples with 6.25 and 12.5 % coverage ratios show a negative differential resistance (NDR) characteristic between 0.2 and 0.4 V. Beyond 0.4 V of applied voltage, rGO with 6.25 % coverage ratio shows non-conducting characteristics, while the rGO with 12.5 % coverage has a constant current behaviour. On the other hand, it was shown in previous studies, that rGO with 50 % coverage would show non-conducting characteristics at zero-bias [21]. The *I*–*V* behaviour obtained in this study also supports this prediction for applied voltages around zero-bias. However, as the applied voltage increases, rGO with 50 % coverage also shows a conducting behaviour as it can be seen from Fig. 2.

It is well-known that the *I*–*V* characteristics of ballistic nano devices stem from the voltage-dependent variation of their transmission spectra [51, 53]. The current, *I*, of a nanodevice is given as in Eq. (1) where *T*(*E*) is the transmission spectrum, *µ*_*R*_ and *µ*_*L*_ are the electrochemical potentials of the right lead and the left lead, respectively, *q* is the elementary charge and *h* is the Planck’s constant. *f*_*L*_(*E*) and *f*_*R*_(*E*) are the Fermi–Dirac functions of the left lead and the right lead, respectively [53].

1 $$I=\frac{2q}{h}\int_{\mu_{R}}^{\mu_{L}}T\left(E\right)\left[f_{L}\left(E\right)-f_{R}\left(E\right)\right]dE.$$

**Fig. 3:**
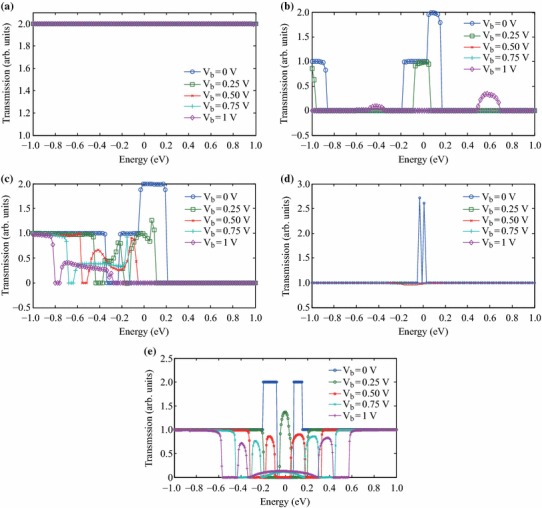
Transmission spectrum of: **a** intrinsic graphene sheet, **b** rGO with 6.25 % coverage, **c** rGO with 12.5 % coverage, **d** rGO with 25 % coverage, **e** rGO with 50 % coverage for various applied voltages

**Fig. 4:**
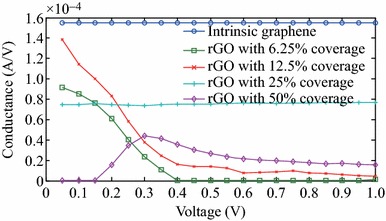
Variations of the conductances of the graphene sheet and rGO samples by the applied voltage

The transmission spectra of the considered graphene and rGO samples are also obtained and plotted in Fig. 3 for various applied voltages. In order to keep the plots clear, the transmission spectra are shown only for the voltages of 0, 0.25, 0.5, 0.75 and 1 V. The transmission spectrum of the intrinsic graphene sheet is constant and almost independent of the applied voltage as shown in Fig. 3a. This leads to its linear *I*–*V* characteristics as given in Fig. 2. On the other hand, for the rGO sample with 6.25 % coverage ratio, while the transmission spectrum is nonzero for about 0 V applied voltage, the transmission characteristic gets narrower for 0.25 V bias voltage as can be seen from Fig. 3b triggering NDR characteristics. As the voltage takes the values of 0.5 and 0.75 V, the transmission spectrum has the value of zero. However, as the applied voltage is further increased, transmission spectrum again starts to have nonzero values in the applied voltage range hence a very small current passes between 0.9 and 1 V as shown in Fig. 2. The behaviour of the transmission spectrum of rGO sample with 12.5 % coverage is also similar to that of the rGO sample with 6.25 % coverage. The transmission spectrum reduces as the applied voltage is increased from 0 to 0.25 V as shown in Fig. 3c causing NDR behaviour. However, the difference of the transmission spectrum of the rGO sample with 12.5 % coverage is that there is always a nonzero region as the applied voltage is varied from 0.4 to 1 V hence the current is nonzero in this voltage range for this sample. The situation is fairly different for the rGO sample with 25 % coverage. According to [21], rGO with 25 % coverage is expected to have conducting characteristics around zero-bias and the transmission spectrum shown in Fig. 4d supports this. However, the important point about the transmission spectrum of the rGO with 25 % coverage is that the transmission value of 1 is maintained across the applied voltage range as the bias voltage increases from 0 to 1 V. This is also seen as a linear *I*–*V* relationship shown in Fig. 2. It is worth noting that the slopes of the *I–V* characteristics of the intrinsic graphene sheet and the rGO sample with 25 % coverage are different originating from different transmission values of Fig. 3a and d, namely the transmission coefficients of the pure graphene sheet are the double of that of the rGO sample with 25 % coverage. The behaviour of the transmission spectrum of the rGO sample with 50 % coverage is also peculiar as seen in Fig. 3e. The transmission spectrum has zero value for the applied voltage range for around 0 V bias voltage. However, as the applied voltage increases, the nonzero part of the transmission spectrum starts to lie in the applied voltage range hence a current starts to flow. From Fig. 2, the *I*–*V* characteristics seems to have a threshold value of 0.15 V for the rGO sample with 50 % coverage.

The conductance–voltage (*G*-*V*) characteristics of the intrinsic graphene sheet and rGO samples are also calculated as shown in Fig. 4. The conductance of the intrinsic graphene sheet is twice of the conductance quantum maintained in the whole voltage range as expected [1]. In addition, although rGO samples with 6.25, 12.5 and 25 % coverages are conducting near zero-bias, their conductances are lower than that of the intrinsic graphene sheet. This is due to the contamination and slightly modified crystal structures of rGO samples which are different from the ideal honeycomb configuration of the intrinsic graphene. The conductance of the rGO sample with 25 % coverage has the value of the conductance quantum since its transmission is 1 as seen on Fig. 3d. On the other hand, the conductances of the rGO samples with 6.25 and 12.5 % coverage show decreasing trend as the applied voltage increases. Moreover, while the rGO sample with 50 % coverage is non-conducting near zero bias, it seems to show conducting characteristics as the applied voltage increases.

**Fig. 5:**
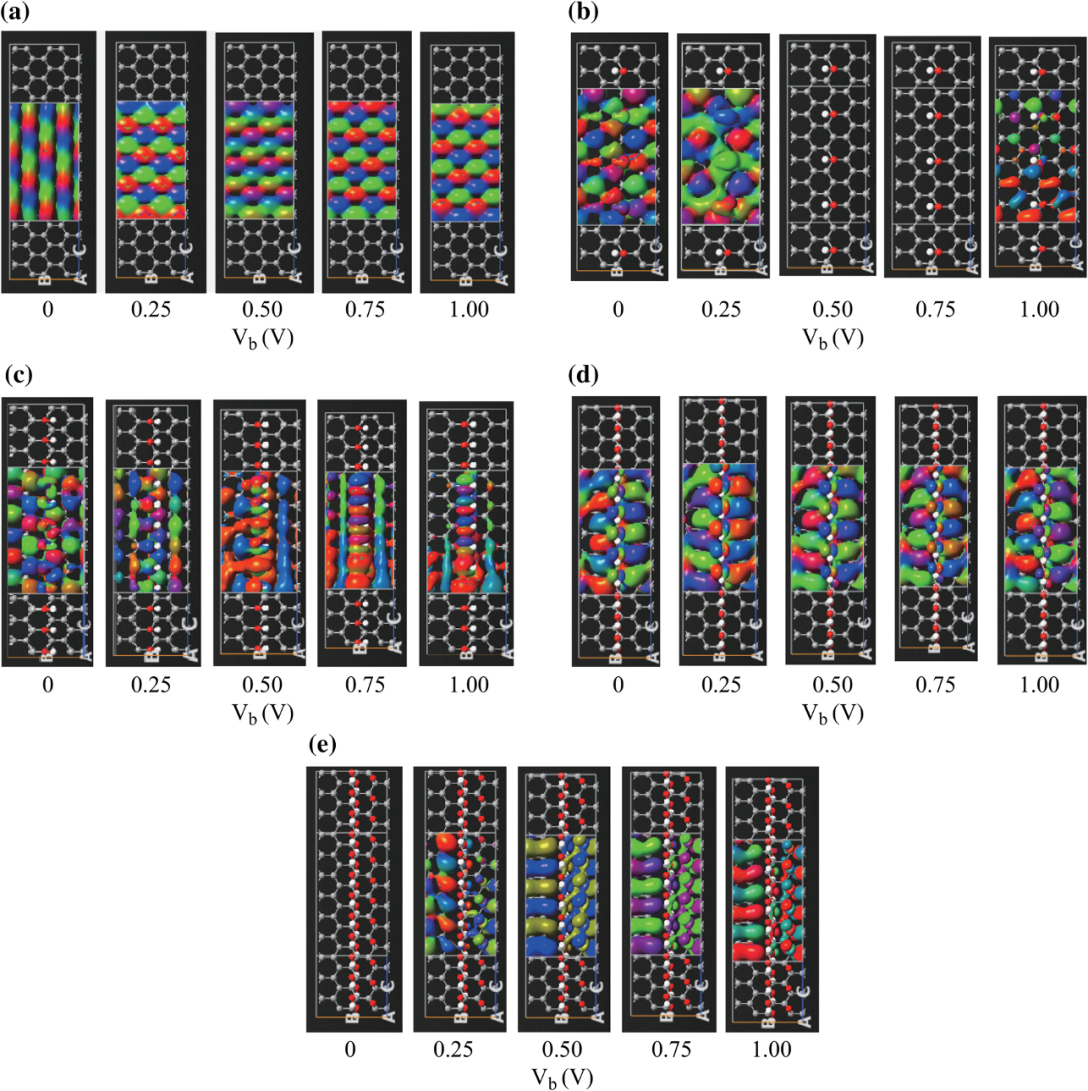
Transmission eigenstates of: **a** intrinsic graphene sheet, **b** rGO with 6.25 % coverage, **c** rGO with 12.5 % coverage, **d** rGO with 25 % coverage, **e** rGO with 50 % coverage for various applied voltages

In order to further investigate the origins of the voltage-dependent conductance characteristics of the rGO samples, the transmission eigenstates are considered. Transmission eigenstates are the linear combination of the scattering states which correspond to the eigenstates of the transmission matrix [54]. Transmission eigenstates practically provide information on the electronic states which contribute to the conduction in a nanostructure. In other words, when a transmission eigenstate exists in a particular region of the structure, it means that conducting electrons can exist in these states. Hence, an electron entering the nanostructure from a lead needs to follow a path induced by the transmission eigenstates. In this context, the localization degree of the transmission eigenstates determines the transmission probability of an electron to pass through the structure. Delocalized eigenstates implies a high electron flow probability while localized eigenstates shows that this probability is low [51, 54]. The variation of the transmission eigenstates is plotted in Fig. 5 for the considered rGO samples and the reference graphene sheet. The transmission eigenstates of the reference graphene sheet is delocalized independent of the applied voltage as shown in Fig. 5a, which is the reason of maintaining the conductance of the graphene sheet at a constant value. The analyses of these plots reveal that the transmission eigenstates of rGO samples with 6.25 and 12.5 % coverage ratios are delocalized near zero bias as can be seen from Fig. 5b and c, respectively. However, as the applied voltage increases, the eigenstates tend to localize which explains the decreasing trend of the *G*-*V* characteristics of these samples. The transmission eigenchannels of the rGO sample with 6.25 % coverage diminish between 0.4 and 0.9 V where the current is zero; therefore, there are no transmission eigenstates appearing for the applied voltages of 0.5 and 0.75 V in Fig. 5b. On the other hand, it can be seen from Fig. 5c that the transmission eigenstates of the rGO sample with 12.5 % coverage ratio do not completely diminish but are relatively localized as the applied voltage increases; therefore, there is always a nonzero current in the 0–1 V range as seen from Fig. 2. Figure 5d shows that the localization degree of the eigenstates of the rGO with 25 % coverage ratio does not change considerably in the 0–1 V voltage range hence this sample shows a highly linear *I*–*V* characteristic. On the other hand, the transmission eigenstates of the rGO sample with 50 % coverage shown in Fig. 5e start to form and delocalize as the applied voltage increases which causes the current of this sample to have nonzero values for bias voltages higher than 0.15 V as given in Fig. 2.

## Conclusions

Considering the reduction of GO is an effective method to implement conducting rGO structures, voltage-dependent transport properties of rGO samples with different coverage ratios are investigated in this study. Electronic transport behaviours of rGO samples with coverages of 6.25, 12.5, 25 and 50 % are obtained together with the properties of intrinsic graphene sheet for comparison. It is exposed that depending on the coverage ratio, rGO structures show different and peculiar electronic transport characteristics such as (i) rGO with 6.25 and 12.5 % coverages show NDR behaviour, (ii) the transmission of the GO sample with 6.25 % coverage ratio diminishes after the NDR voltage range, (iii) the *I*–*V* characteristic of the rGO with 25 % coverage is highly linear making it to be suitable for use as interconnects and (iv) while rGO with 50 % coverage shows non-conducting behaviour near zero applied voltage as estimated by previous studies [21], it starts to be conducting beyond a threshold voltage. These different transport characteristics of rGO samples are shown to be originated from the variation of their transmission eigenstates and the localization degree of these eigenstates by the applied voltage. It is concluded that the voltage-dependent transport properties of rGO structures have diverse behaviours depending on the coverage ratio hence their peculiar *I*–*V* characteristics can be engineered to achieve specific functions in nanoscale circuits easily since the coverage ratios of rGO structures can be precisely controlled using reduction by laser light.
